# Fluorophore placement and size impact E2 ubiquitination efficiency

**DOI:** 10.1042/BCJ20260496

**Published:** 2026-10-08

**Authors:** Jashanjot Kaur Gill, Rachel E. Lacoursiere, Gary S. Shaw

**Affiliations:** Department of Biochemistry, The University of Western Ontario, London, Ontario, Canada, N6A 5C1

**Keywords:** enzyme activity, fluorescence resonance energy transfer, ubiquitins

## Abstract

E2 conjugating enzymes function in the ubiquitin-mediated proteolysis pathway by accepting ubiquitin (Ub) from an E1 enzyme (Uba1), forming an E2∼Ub thioester conjugate, and transferring Ub to a substrate or to an E3 ligase enzyme. Fluorescent or affinity tags are often used as sensitive readouts for catalytic variability and specificity of E2 enzymes and their variants. Here, we examined how the size or placement of fluorescent tags compromise Ub conjugation for the E2 enzymes UbcH7, UbcH5a, and Ubc13. Large green fluorescent protein (GFP)-based fluorophores were placed at the N- or C-terminus of the E2 enzyme UbcH7 and the N-terminus of Ub, and compared with Alexa-based dyes, approximately 30 times smaller, at the same positions. While all modified proteins formed E2∼Ub conjugates, placing a smaller Alexa dye at the C-terminus of the E2 enzyme improves the initial rate of E2∼Ub conjugate formation by up to 10-fold compared with a larger GFP-based tag (YPet) at the N-terminus. In the case of UbcH5a, YPet labelling at its N-terminus significantly slowed formation of an E2∼Ub conjugate and Ub unloading to the HECT domain of HUWE1 when compared to UbcH7. Overall, we find that small Alexa-based tags, especially when placed at the C-terminus of E2 enzymes, are an optimal choice for monitoring E2 ubiquitination using fluorescence-based experiments.

## Introduction

Ubiquitination is a dynamic post-translational modification (PTM) where the small protein ubiquitin (Ub) is continuously added and removed from cellular proteins as a signal for DNA damage repair, proteasomal degradation, cell cycle progression and other processes [1–3]. The ubiquitination cascade involves the adenylation of Ub and subsequent formation of a Ub-thioester with an E1 activating enzyme, followed by transthiolation to an E2 conjugating enzyme to form an E2∼Ub conjugate [4]. Multiple categories of E3 enzymes act as conduits to transfer Ub from an E2∼Ub conjugate to a substrate protein. Included in this group are the RING (Really Interesting New Gene), HECT (Homologous to E6-AP C-terminus) and RBR (RING-inBetween-Rcat) E3 ligases that differ in their modes of Ub transfer. The ubiquitination process is complex due to the number of E2 (∼40) and E3 (∼600) enzymes available that provide substrate diversity and selectivity [5]. Canonical ubiquitination occurs at lysine residues of substrates, although other types of modifications are possible [6,7]. Not surprisingly, defects in the ubiquitination pathway are associated with various cancers, neurodegenerative diseases and immune disorders [8–11].

E2 conjugating enzymes are the key intermediaries in the ubiquitination cascade because they recruit and transfer Ub between E1 activating enzymes and E3 ligase enzymes and substrates [12–14]. Ub transfer through the ubiquitination cascade is promoted by the coupling of adenylation and transthiolation reactions catalyzed by an E1 enzyme to form the E2∼Ub conjugate [15] and the higher affinity for an E2∼Ub conjugate compared with the unloaded E2 enzyme for E3 ligases [16,17]. E2 enzymes are also a major driver of ubiquitin chain linkage type when paired with specific RING E3 ligases [18]. For example, E2 enzymes, such as UBE2S and UBE2R1, form Lys11 and Lys48-linked ubiquitin chains with the anaphase-promoting complex (APC/C) and Skp1-cullin-F-box (SCF) E3 enzymes, respectively [19,20]. Each E2 enzyme contains a structurally conserved, ubiquitin-conjugating (UBC) domain comprising four α-helices (α1–α4), five β-strands (β1–β5) and an essential catalytic cysteine residue that forms a thioester intermediate with Ub. Crystal structures show that basic residues (K/RRXXK/R) within the N-terminal α-helix (α1) of E2 enzymes, for example, CDC34 (Arg14, Arg17) and Ubc15 (Lys12, Lys15), interact with a conserved acidic patch in the ubiquitin-fold domain (UFD) of the E1 Uba1 (Figure 1) [21–25]. Upon receiving Ub from an E1 enzyme, the E2∼Ub conjugate adopts a dynamic range of conformations with endpoints corresponding to the E2 and Ub in compact (“closed”) and more extended (“open”) arrangements recognized by RING E3 ligases and HECT/RBR E3 ligases, respectively [26–28]. This is particularly evident for the E2 enzyme UbcH7, which favors a closed E2∼Ub conformation in the absence of an E3 enzyme and rearranges to an open conformation upon complex formation with HECT or RBR E3 ligases, optimizing Ub transfer to the catalytic cysteine residue on these enzymes [28,29].

**Figure 1:**
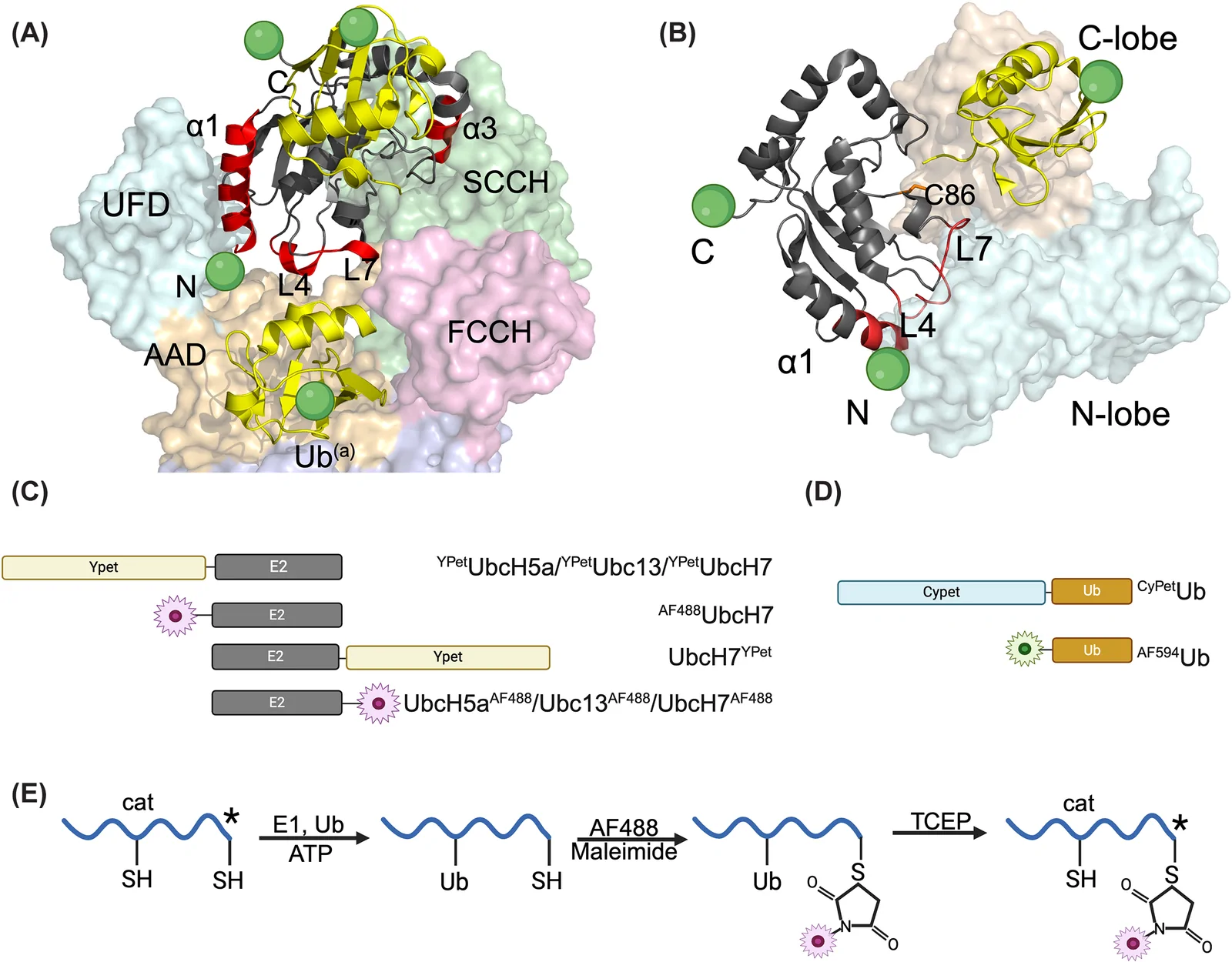
Design and fluorophore labelling strategy for E2 and Ub proteins (**A**) Model of an E2∼Ub conjugate with Ub-adenylate loaded E1 enzyme Uba1. The model was created by superimposing the structure of UbcH7 (grey) (PDB: 6XXU, conformer 1) [81] with the E2 enzyme Ubc4 from the structure of double Ub-loaded E1 with Ub at the donor position in the E1–E2 Ub transthiolation step (PDB: 9B5E) [15]. The major interacting areas of the E2 enzyme (α1 helix, α3 helix, and loops L4 and L7) are shown in red. Ubiquitin molecules are shown in yellow. The E1 domains interacting with E2 and Ub are the ubiquitin fold domain (UFD) (blue), active adenylation domain (AAD) (wheat), first catalytic cysteine half-domain (FCCH) (pink) and second catalytic half-domain (SCCH) (green). The position of fluorophores on UbcH7 (N and C-termini) and Ub (N-terminus) constructs are shown as green spheres. (**B**) Model of a UbcH7∼Ub conjugate with a HECT E3 ligase. The model was created by superimposing the structure of UbcH7 (grey) (PDB: 6XXU, conformer 1) with UbcH5c in the structure of UbcH5c∼Ub with the HECT E3 ligase NEDD4L (surface) (PDB: 3JW0) [42]. The interacting areas of the E2 enzyme (α1 helix, and loops L4 and L7) are shown in red. (**C**) FRET construct design for UbcH7 showing YPet or AF488 fluorophores at the N- and C-termini of the E2 enzyme. ^YPet^UbcH5a, ^YPet^Ubc13, UbcH5a^AF488^, and Ubc13^AF488^ constructs were similarly designed. (**D**) CyPet or AF594 fluorophores at the N-terminus of Ub. (**E**) AF488 fluorescent labelling strategy. Constructs with either an N-terminal or C-terminal cysteine residue (*) were incubated with Uba1, ATP and Ub to form E2∼Ub conjugates using the catalytic cysteine (cat). AF488-maleimide was then added for site specific fluorescent labelling of the E2 at either the N- or C-terminus followed by reduction to obtain either the ^AF488^E2 or E2^AF488^ proteins (see Materials and methods section for details).

The efficiency of Ub transfer is most notable in experiments that capture E1∼Ub, E2∼Ub or E3∼Ub thioester intermediates that form rapidly during *in vitro* reactions but can also quickly dissolve during measurements. This has prompted the use of ester, isopeptide, disulfide and other linkages to stabilize enzyme–Ub complexes for structural studies [30]. Many early studies utilized different E2 enzymes interchangeably in ubiquitination reactions, frequently to observe qualitative E2∼Ub conjugate formation or substrate ubiquitination [25]. However, multiple studies show that precise kinetic measurements (*k*_cat_, *K*_m_) reveal quantitative differences resulting from the choice of E1 enzyme, type of Ubl or minor substitutions in any of the ubiquitination components [31–33]. Traditionally, methods such as radio-labelled ubiquitin coupled with gel-based assays have successfully measured the initial Ub transfer between E1 and E2 enzymes [16,34,35]. Alternate methods that use fluorescently tagged enzymes are available [36–38], although kinetic experiments indicate the addition of large fluorophores may alter the reaction kinetics likely due to steric hindrance during the formation of the E1–E2 or E2–E3 transthiolation complexes [39,40]. In this work, we investigated the impact of fluorophore size and location on the efficiency of Ub transfer from an E1 enzyme to an E2 enzyme and unloading of a E2∼Ub conjugate by a HECT E3 ligase, using real-time Förster Resonance Energy Transfer (FRET) measurements. We show that fluorophores at the N-terminus of an E2 enzyme have a strong influence on E2~Ub conjugate formation and should be replaced with smaller benign reporters at the C-terminus of the E2 enzyme. The use of sensitive Alexa fluorophores allows quantitative loading and unloading activity of E2 enzymes to be monitored in real time with minimal interference and has the potential to explore small differences in kinetics due to substitutions and PTMs in the ubiquitination pathway.

## Results

### Design of fluorophore-labelled E2 and Ub proteins

Helix α1, the L4 and L7 loop regions and the N-terminus of helix α3 of an E2 enzyme interact with UFD, AAD and SCCH domains of an E1 enzyme (Figure 1A) [21–24,41]. Similarly, an E2∼Ub conjugate uses the N-terminal region of helix α1 and loops L4 and L7 to recruit the N-lobe of a HECT E3 ligase while the conjugated Ub interfaces with the C-lobe of the E3 enzyme (Figure 1B) [42]. This latter arrangement positions the E2∼Ub conjugate in an open conformation, placing the catalytic cysteine residues of the two enzymes in close proximity, thereby facilitating Ub transfer (Figure 1B) [42]. RBR E3 ligases also utilize an open E2∼Ub conformation facilitated by multiple domains in the E3 ligase to facilitate Ub transfer to a catalytic cysteine residue in the catalytic Rcat domain [28,29]. In RING E3 ligases, helix α1 and loops L4 and L7 of the E2 enzyme interact with the zinc coordinating loops L1 and L2 along with the central α5 helix regions of the RING domain while the conjugated ubiquitin associates with the E2 enzyme in the closed conformation [43–46]. These observations from structures of E1/E2 and E2/E3 complexes indicate that the N-terminus of an E2 enzyme has significant interactions with its partner enzymes, while its C-terminus has a more limited role. We tested how N- and C-terminal modifications might impact Ub transfer from an E1 enzyme to the E2 conjugating enzymes UbcH7, UbcH5a and Ubc13. Additionally, we examined Ub unloading from an E2∼Ub conjugate to a HECT E3 enzyme, utilizing the E2 enzymes UbcH7 and UbcH5a that function efficiently in this pathway [47–49]. E2 constructs were designed with large GFP-based donor fluorophores (YPet or CyPet) placed at either the N- or C-terminus of UbcH7 (^YPet^UbcH7, UbcH7^YPet^), or N-terminus of UbcH5a (^YPet^UbcH5a), Ubc13 (^YPet^Ubc13) and Ub (^CyPet^Ub). Smaller Alexa-based dyes (Alexa Fluor 488–AF488 or Alexa Fluor 594–AF594) were added to the N- or C-terminus of UbcH7 (^AF488^UbcH7, UbcH7^AF488^), C-terminus of UbcH5a (UbcH5a^AF488^) or Ubc13 (Ubc13^AF488^) and the N-terminus of Ub (^AF594^Ub) (Figure 1C,D). The Alexa-based dyes are approximately 30 times smaller (∼1 kDa) than traditional GFP-based fluorophores with comparable quantum yields and extinction coefficients. This approach allowed 12 different paired combinations to be assessed using FRET-based ubiquitination assays to identify how the position and size of the fluorophores might alter ubiquitin transfer.

GFP-based fluorophores (YPet, CyPet) were incorporated into E2 enzymes and Ub as previously described [40,50]. ^AF594^Ub was prepared by labelling a Ub construct, containing an added N-terminal cysteine [51], with AF594 using maleimide chemistry (Supplementary Figure S1) [52]. To label E2 enzymes with AF488 we used two strategies, highlighted for UbcH7. The first approach used cycloaddition-based click chemistry [53] through the incorporation of azidohomoalanine (AHA, a methionine azide analog) at either the N- or C-terminal methionine positions of UbcH7 and copper-free catalysis to append AF488 fluorophores (Supplementary Figure S2). Although this method successfully added AF488 to the UbcH7 C-terminus, incorporation at the N-terminus resulted in irreversible reduction of the AHA azido group, previously observed in other systems [54], leading to incomplete AF488 labelling and mixed fluorescently labelled UbcH7 products (Supplementary Figure S2). Instead, we devised a thiol-based maleimide strategy that involved adding an additional cysteine residue to either the N- or C-terminus of UbcH7 and enzymatically forming the respective UbcH7~Ub conjugates using Uba1 and Mg^2+^/ATP. Subsequent reaction with AF488 maleimide to label the free N- or C-terminus, followed by reduction of the UbcH7~Ub conjugate, yielded site-specific fluorescent-labelled ^AF488^UbcH7 or UbcH7^AF488^ while keeping the catalytic C86 available for future conjugation reactions (Figure 1E and Supplementary Figure S1). A similar approach was used to label UbcH5a and Ubc13 with AF488 at their C-termini.

### Properties of the FRET E2~Ub pairs

Gel-based loading assays were initially used to observe the formation of E2~Ub conjugate complexes (Figure 2) as illustrated for UbcH7. At 10 min, all eight combinations of the fluorescently labelled UbcH7 (^YPet^UbcH7, UbcH7^YPet^, ^AF488^UbcH7, UbcH7^AF488^) and Ub (^CyPet^Ub, ^AF594^Ub) proteins show formation of the appropriate E2~Ub conjugate with the expected ∼9 kDa shift upon addition of ^AF594^Ub and ∼38 kDa shift upon addition of ^CyPet^Ub. The formation of each E2~Ub conjugate was confirmed through reduction of the thioester bond with DTT, restoring the individual E2 and Ub proteins (Figure 2). These assays demonstrated that all fluorescently labelled E2 and Ub combinations are functional. Some minor differences in the efficiency of E2~Ub conjugation are noted when comparing selected E2/Ub pairs. However, the experiment is more compromised by large differences in protein positions on the gel and by differences in protein staining properties, most evident by the intensity differences between YPet- and AF488-labelled UbcH7 constructs. In addition, sensitivity issues made lower concentrations more difficult to measure reproducibly.

**Figure 2:**
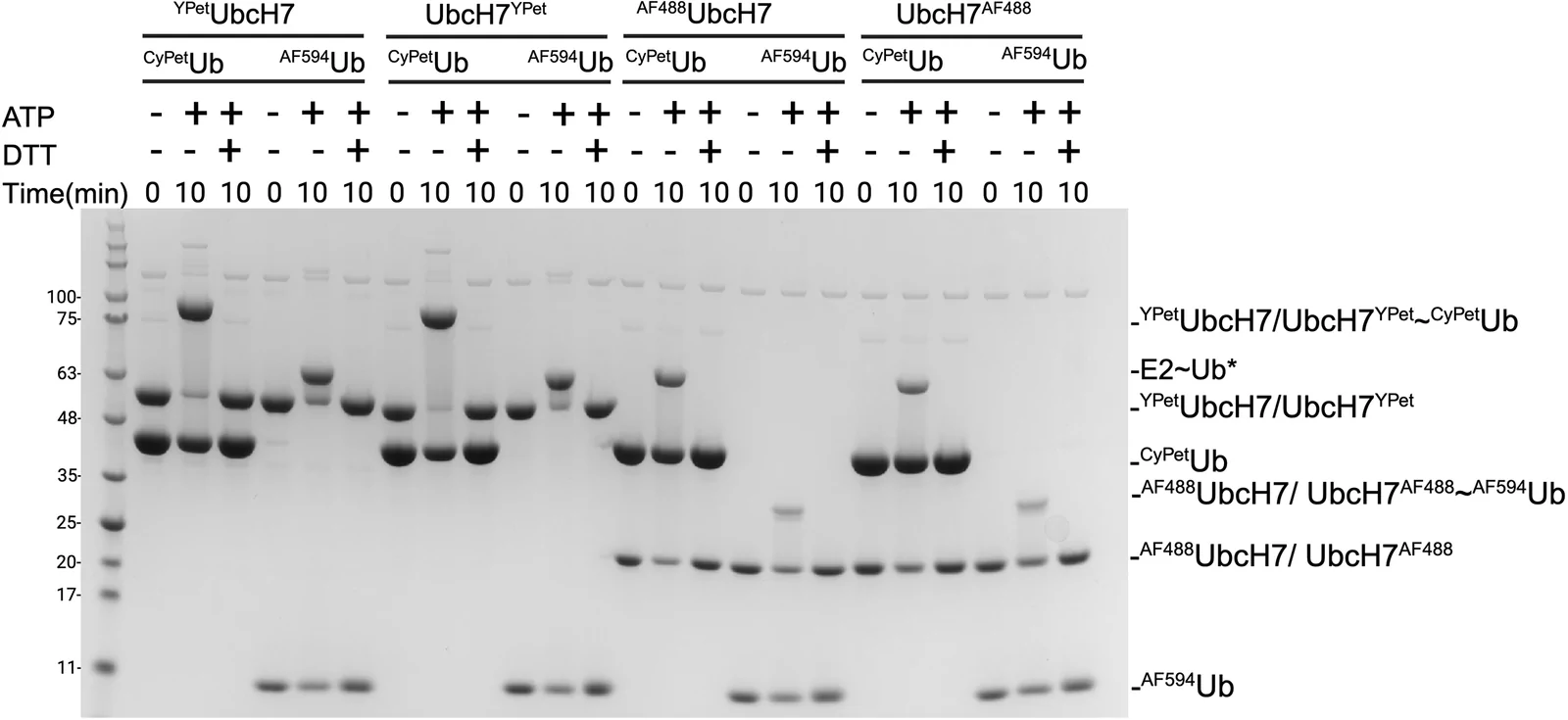
Fluorescent E2 and Ub proteins form E2∼Ub conjugates Formation of E2∼Ub conjugates monitored by SDS–PAGE. The four UbcH7 fluorescent constructs (^YPet^UbcH7, UbcH7^YPet^, ^AF488^UbcH7 and UbcH7^AF488^) were incubated in the presence of Uba1 and each of the two Ub constructs (^CyPet^Ub and ^AF594^Ub) in the absence and presence of ATP for 10 min. Bands representing the formation of each E2~Ub conjugate using ^CyPet^Ub (^YPet^UbcH7/UbcH7^YPet^–^CyPet^Ub, ^AF488^UbcH7/UbcH7^AF488^–^CyPet^Ub) or ^AF594^Ub (^AF488^UbcH7/UbcH7^AF488^–^AF594^Ub, ^YPet^UbcH7/UbcH7^YPet^∼^AF594^Ub) are labelled. ^AF488^UbcH7/UbcH7^AF488^–^CyPet^Ub and ^YPet^UbcH7/UbcH7^YPet^–^AF594^Ub are labelled as E2~Ub*. Following each reaction all E2~Ub conjugates were reduced with DTT to confirm thioester conjugate formation.

To measure the formation of E2~Ub conjugates using real-time fluorescence measurements, the compatibility of fluorescent E2 and Ub constructs as FRET pairs was assessed. Initially, four donor–acceptor FRET pairs for UbcH7 and Ub were used in this study, namely, CyPet–YPet, YPet–AF594, CyPet–AF488 and AF488–AF594, chosen due to the overlap between the donor emission spectra and the acceptor excitation spectra (Supplementary Figure S3). For example, in the CyPet–YPet pair, ^CyPet^Ub is excited at 420 nm and the emission of either ^YPet^UbcH7 or UbcH7^YPet^ construct is measured at 528 nm upon E2~Ub conjugate formation, as previously described [50,55]. Upon reaction initiation with Uba1 and ATP, a decrease in donor CyPet emission is observed coincident with an increase in acceptor YPet fluorescence due to the formation of the ^YPet^E2–^CyPet^Ub conjugate (Figure 3A). All other FRET pairs give large similar increases in acceptor fluorescence signal upon formation of their E2~Ub conjugates (Supplementary Figure S3 and Supplementary Table S1). Obvious differences are noted for the maximum FRET signal with respect to the location of the fluorophore. For example, formation of an E2~Ub conjugate with the YPet fluorophore at the C-terminus of UbcH7 (UbcH7^YPet^–^CyPet^Ub) yields a FRET signal approximately 250% larger than the N-terminal location using the same donor fluorophore for Ub (^YPet^UbcH7–^CyPet^Ub) (Figure 3A). Qualitatively, this indicates the fluorophore pairs are approximately 5 Å closer (on average) in the E2~Ub conjugate containing the C-terminal UbcH7^YPet^ based on the Förster relationship.

**Figure 3:**
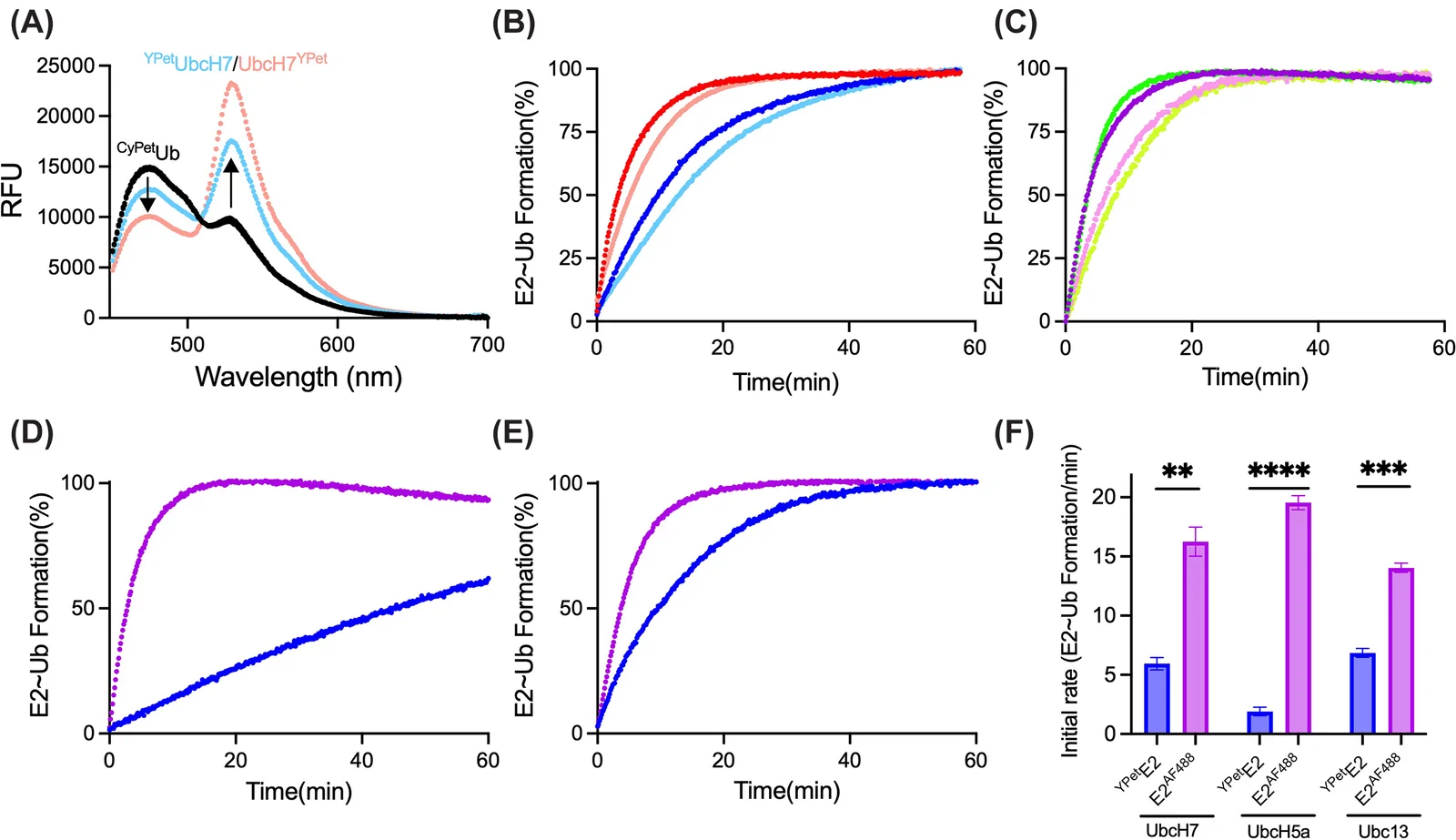
E2~Ub conjugate formation is sensitive to fluorophore placement and size Emission spectra and E2~Ub conjugate formation measured using FRET. (**A**) Emission spectra for ^CyPet^Ub at time zero (black) and after 30 min upon formation of an E2~Ub conjugate with either ^YPet^UbcH7 (cyan) and UbcH7^YPet^ (salmon). (**B**) Normalized formation of UbcH7~Ub conjugates for equimolar amounts of E2 and Ub using ^YPet^UbcH7 with ^CyPet^Ub (cyan) or ^AF594^Ub (blue), and UbcH7^YPet^ with ^CyPet^Ub (pink) and ^AF594^Ub (red). (**C**) Normalized formation of UbcH7~Ub conjugates for equimolar amounts of E2 and Ub using ^AF488^UbcH7 with ^CyPet^Ub (light green) or ^AF594^Ub (green), and UbcH7^AF488^ with ^CyPet^Ub (pink) or ^AF594^Ub (purple). (**D**) Normalized formation of UbcH5a~Ub conjugates for equimolar amounts of E2 and Ub using ^YPet^UbcH5a with ^AF594^Ub (blue) and UbcH5a^AF488^ with ^AF594^Ub (purple). (**E**) Normalized formation of Ubc13~Ub conjugates for equimolar amounts of E2 and Ub using ^YPet^Ubc13 with ^AF594^Ub (blue) and Ubc13^AF488^ with ^AF594^Ub (purple). (**F**) Comparison of E2~Ub initial rates of formation for UbcH7, UbcH5a and Ubc13 using ^AF594^Ub and either ^YPet^E2 or E2^AF488^ based on data shown in panels (B–E). A Student’s *t*-test was used to perform statistical analysis for *P*-values ≤ 0.01 (**), 0.001 (***), and 0.0001 (****).

### Fluorophore location and size alter initial rates of E2~Ub conjugate formation

To assess how placement and size of the different fluorophores affect E2 loading, we measured the rates of formation for each E2~Ub conjugate using the eight FRET pair combinations for UbcH7 and a subset of FRET combinations for UbcH5a and Ubc13. Rates were measured using the increase in the acceptor fluorescence as a function of time and normalized for complete conjugate formation. All rate curves show a hyperbolic shape that maximizes upon complete formation of the different E2~Ub conjugates (Figure 3). In some cases, a slowly-decaying decrease in the maximum FRET signal is observed at extended measurement times, likely due to hydrolysis or back transfer to the E1 enzyme as previously observed [56–58].

Marked differences in the rates of E2~Ub formation are noted when the large GFP-type fluorophore (YPet) is moved from the N-terminus to the C-terminus of the UbcH7 enzyme. For example, the initial rate of UbcH7^YPet^~^CyPet^Ub conjugate formation is about two times faster than ^YPet^UbcH7~^CyPet^Ub (Figure 3B, pink versus cyan curves). For either GFP position on UbcH7, the initial rate is also increased when the donor fluorophore on Ub is swapped to the smaller AF594 dye (red versus blue data). These trends are also apparent using a smaller AF488 dye on the E2 enzyme and either CyPet or AF594 at the N-terminus of Ub. In these cases, an approximate 2-fold enhancement for the initial rate of conjugate formation occurs when the donor Ub fluorophore size is decreased from CyPet to AF594 regardless of whether the AF488 fluorophore is at the N-terminus of UbcH7 (^AF488^UbcH7, light green versus green) or C-terminus (UbcH7^AF488^, pink versus purple) (Figure 3C). These data show that the extremities for E2~Ub formation are a large YPet fluorophore at the N-terminus (slowest) and a small AF488 fluor at the C-terminus (fastest) of UbcH7. To test this idea with other E2 enzymes, we assessed these fluorophores in E2~Ub conjugation assays using UbcH5a and Ubc13 with ^AF594^Ub. Consistent with the UbcH7 observations, these experiments (Figure 3D–F) show an increase in the initial rate of E2~Ub formation, ranging from 2-fold (Ubc13) to 10-fold (UbcH5a), is achieved using the C-terminal E2^AF488^ proteins compared to the N-terminal YPet constructs (^YPet^UbcH5a, ^YPet^Ubc13). These observations indicate that the location and size of the fluorophore on an E2 enzyme and the size of the fluorophore on Ub have significant influences on the overall efficiency of E2~Ub conjugate formation.

### Large fluorescent tags impair kinetics of UbcH7~Ub conjugate formation

To better quantify the impact that fluorescent tag position and size have on E2~Ub conjugate formation we measured Michaelis–Menten kinetics for eight FRET pair combinations using UbcH7 as a representative E2 enzyme. Our approach allowed fluorescent measurements to be rapidly acquired (1 point/10 s) to determine the initial rates of reaction for a variety of E2 concentrations (Figures 3 and 4). We compared E2~Ub loading kinetics using the E1 enzyme Uba1 pre-loaded with Ub having two different sizes of fluorophore at the N-terminus, ^CyPet^Ub and ^AF594^Ub, with the E2 substrate constructs ^YPet^UbcH7, UbcH7^YPet^, ^AF488^UbcH7 and UbcH7^AF488^. This approach quantified the catalytic and affinity differences observed by moving the fluorophore from the N-terminus of UbcH7 to its C-terminus or replacing a bulky GFP-based fluorophore with a smaller Alexa dye for both UbcH7 and Ub (Figure 4).

**Figure 4:**
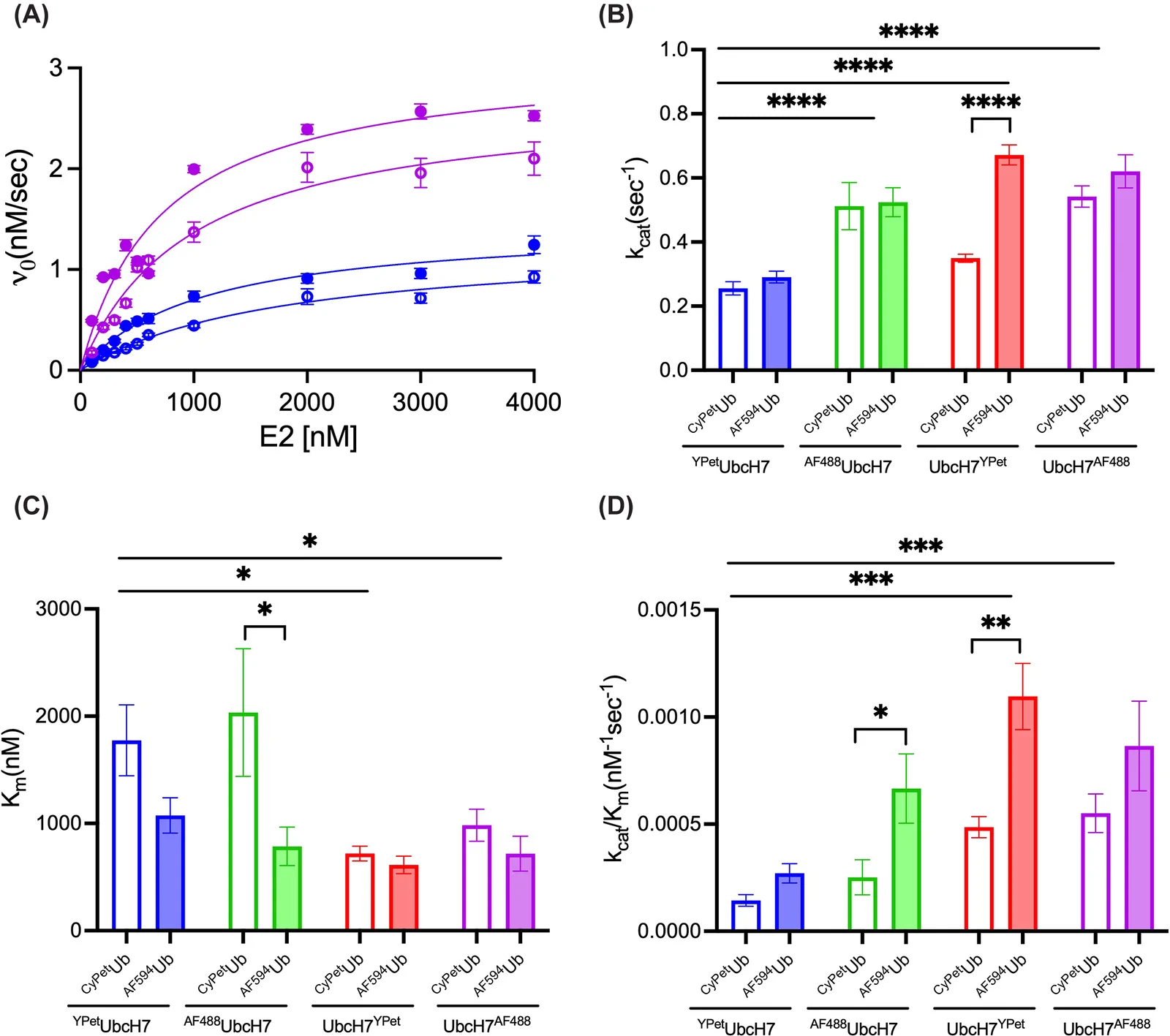
Kinetics of E2~Ub conjugate formation using different FRET pairs for UbcH7 (**A**) Michaelis–Menten curves for E2 loading of ^YPet^UbcH7 (blue) or UbcH7^AF488^ (purple) with either ^CyPet^Ub (empty circles) or ^AF594^Ub (filled circles). The initial rates for E2~Ub product formation (*ν*_0_) are plotted for a range of E2 concentrations. Each point represents an average of three independent replicates with standard error of the mean bars. (**B**) *k*_cat_, (**C**) *K*_m_ and (**D**) *k*_cat_/*K*_m_ determinations for E2~Ub conjugate formation kinetics for four different E2 constructs ^YPet^UbcH7 (blue), ^AF488^UbcH7 (green), UbcH7^YPet (^red), and UbcH7^AF488^ (purple) using ^CyPet^Ub (empty bars) and ^AF594^Ub (filled bars). Two-way Anova was used to perform statistical analysis for *P-*values ≤ 0.05 (*), 0.01 (**), 0.001 (***) and 0.0001 (****).

Examination of the kinetic data for UbcH7 shows a wide range of efficiencies for E2~Ub conjugate formation depending on the placement and size of the fluorophores used (Figure 4). As observed in Figure 3, positioning the larger GFP fluorophore at the N-terminus of UbcH7 (^YPet^UbcH7 – blue curves) results in the poorest efficiency for E2~Ub conjugate formation while the use of a smaller AF488 at the C-terminus of UbcH7 (UbcH7^AF488^ – purple curves) results in the most efficient conjugation (Figure 4A). The use of ^YPet^UbcH7 consistently has the lowest reaction rate (defined by *k*_cat_) compared with the rest of the UbcH7 constructs (Figure 4B). This is most pronounced for conjugate formation using the smallest fluorophores at the N-terminus of Ub and C-terminus of UbcH7 (^AF594^Ub~UbcH7^AF488^) that have *k*_cat_ values nearly 300% larger than those for the bulky GFP fluorophores (^YPet^UbcH7~^CyPet^Ub). The results also show that fine tuning of the reaction rates is more evident once the bulky fluorophore at the N-terminus of UbcH7 is modified. Both Ub fluorophores show similar rates of reactivity across the different UbcH7 constructs except for UbcH7^YPet^, where a nearly 200% increase in reaction rate is observed for ^AF594^Ub compared with ^CyPet^Ub. This suggests that the bulky fluorophore E2 constructs (^YPet^UbcH7, UbcH7^YPet^) are more sensitive to changes in the Ub fluorophore size (^AF594^Ub versus ^CyPet^Ub) once the YPet fluorophore is removed from the N-terminus of the E2 enzyme. Switching to the smaller AF488 at the N-terminus of UbcH7 (^AF488^UbcH7) is sufficient to recover reaction kinetics that are similar to those when the fluorophore is placed at the C-terminus of UbcH7 (UbcH7^YPet^, UbcH7^AF488^) irrespective of the Ub construct used.

The N-terminally labelled E2 constructs (^YPet^UbcH7, ^AF488^UbcH7) show apparent binding affinities (as defined by *K*_m_) that are larger than all other fluorophore combinations suggesting that placement of either fluorophore at the N-terminus of the E2 enzyme can affect the interaction with Uba1 (Figure 4C). This observation is unexpected for ^AF488^UbcH7 based on the significantly improved reaction rate (*k*_cat_) compared with ^YPet^UbcH7 and indicates that even a non-bulky fluorophore such as AF488 at the N-terminus of UbcH7 is sufficient to influence E1–E2 interactions. Interestingly, a nearly two-fold improvement in E1–E2 affinity is observed for these N-terminal labelled UbcH7 constructs (^YPet^UbcH7, ^AF488^UbcH7) when ^CyPet^Ub is replaced with the ^AF594^Ub construct. This suggests that a bulky fluorophore at the N-terminus of Ub can negatively impact E2 affinity for Ub-bound Uba1, especially when additional fluorophores are present at the N-terminus of the E2 enzyme. As expected, moving the fluorophores from the N-terminus of UbcH7 to its C-terminus (UbcH7^YPet^, UbcH7^AF488^) yield a three-fold decrease in *K*_m_ independent of the Ub fluorophore used.

Overall, we observed a 2.5–5-fold increase in catalytic efficiency (*k*_cat_/*K*_m_) for the formation of UbcH7~Ub conjugates when switching N-terminal tagged UbcH7 (^YPet^UbcH7, ^AF488^UbcH7) to the enzyme with C-terminal tags (UbcH7^YPet^, UbcH7^AF488^) (Figure 4D). A consistent doubling of the catalytic efficiency is observed when the E2 enzyme is loaded with non-bulky ^AF594^Ub compared with ^CyPet^Ub. Combined, these changes result in nearly a 10-fold improvement of Uba1-catalyzed E2~Ub conjugate formation using the UbcH7^AF488^, ^AF594^Ub FRET pair compared with the ^YPet^UbcH7, ^CyPet^Ub partners. This shows that small tags (AF488, AF594), especially when placed at the C-terminus for UbcH7, significantly improve catalysis for Uba1 catalyzed E2~Ub conjugate formation in comparison with large GFP-based tags.

### Fluorescent tags impact E2 binding with Uba1

Our results indicate that the placement and size of a fluorescent tag, and ultimately any type of protein or molecular appendage, can alter the catalytic efficiency for Uba1 catalyzed E2~Ub conjugate formation. To place this in the context of the unmodified proteins, we investigated the catalytic efficiency of UbcH7~Ub conjugate formation using the four UbcH7 constructs (^YPet^UbcH7, UbcH7^YPet^, ^AF488^UbcH7, UbcH7^AF488^) under competitive inhibition conditions compared to unmodified UbcH7 (Figure 5). As expected, the different UbcH7~Ub conjugates show distinctive dose-dependent decreases in E2~Ub conjugation as measured by the relative rates in the absence and presence of increasing concentrations of UbcH7 (*ν_i_/ν_0_*) (Figure 5A). The most significant inhibition is observed for ^YPet^UbcH7, consistent with our observations that a bulky N-terminal fluorophore impairs loading of Ub to UbcH7 (Figure 5B). In contrast, the smallest fluorophore tested (AF488), placed at the C-terminus of UbcH7 (UbcH7^AF488^), shows the weakest inhibition. This trend is observed for E2 loading with both Ub constructs. Since the *K*_i_ is the apparent *K*_m_ for unmodified UbcH7 in comparison with the FRET-labelled E2 enzymes we used the ratio of the Michaelis constant (*K*_m_) and the measured inhibition constants (*K*_i_) to provide evidence for differences in relative affinity between the FRET-based UbcH7 constructs and unmodified UbcH7 to Uba1. The ratio (*K*_m_/*K*_i_) is highest for the N-terminally labelled UbcH7 constructs (^YPet^UbcH7, ^AF488^UbcH7) and lowest for C-terminally labelled UbcH7 constructs (Figure 5C). The data are most dramatic for ^YPet^UbcH7 that shows 10–20 times lower affinity compared to unmodified UbcH7. Similarly, the affinity of ^AF488^UbcH7 for Uba1 remains about 3–4 times poorer than unlabelled UbcH7. In contrast, the C-terminally labelled UbcH7 constructs (UbcH7^YPet^, UbcH7^AF488^) show Uba1 affinity more in line with unmodified UbcH7. Notably, UbcH7^AF488^ in combination with ^AF594^Ub has a *K*_m_/*K*_i_ near unity indicating similar affinity for Uba1 as unmodified UbcH7. These observations reinforce our kinetics experiments (Figure 4) and combined with our initial E2∼Ub formation experiments (Figure 3) establish UbcH7^AF488^ (also UbcH5a^AF488^ and Ubc13^AF488^) along with ^AF594^Ub as the preferred pair to use in E2~Ub conjugation experiments.

**Figure 5:**
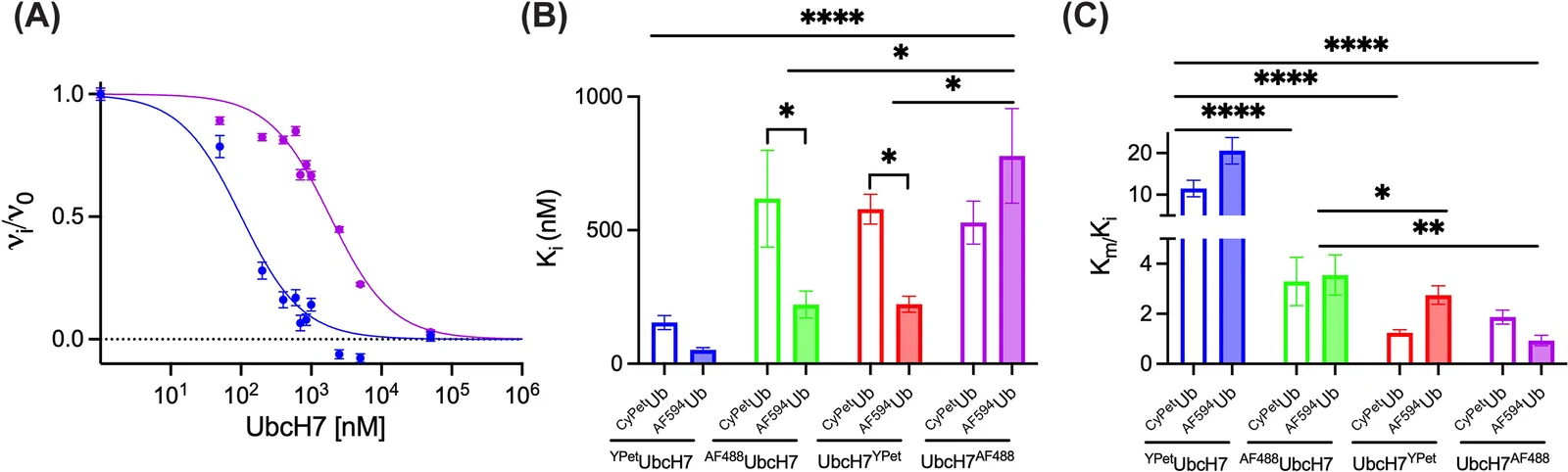
UbcH7^AF488^ has similar affinity for Uba1 as unmodified UbcH7 Dose–response curve for competitive inhibition of FRET tagged E2~Ub conjugate formation using unmodified UbcH7. (**A**) Inhibition of ^YPet^UbcH7∼^AF594^Ub (blue) and UbcH7^AF488^∼^AF594^Ub (purple) conjugate formation for a range of concentrations of unmodified UbcH7 (inhibitor). The data are plotted as the ratio of the observed rate for E2~Ub formation in the presence (*ν_i_*) and absence (*ν_0_*) of inhibitor. Each point represents an average of three independent replicates with standard error to the mean bars. (**B**) Apparent inhibition constants (*K*_i_) determined for E2–Uba1 affinity for FRET E2 constructs: ^YPet^UbcH7 (blue), ^AF488^UbcH7 (green), UbcH7^YPet^ (red) and UbcH7^AF488^ (purple) using either ^CyPet^Ub (empty bars) and ^AF594^Ub (filled bars). (**C**) Ratio of Michaelis constant (*K*_m_) to apparent the inhibition constants (*K*_i_) for Uba1 catalyzed FRET E2~Ub construct conjugate formation in comparison to unmodified UbcH7. Two-way Anova was used to perform statistical analysis for *P-*values ≤ 0.05 (*), 0.01 (**) and 0.0001 (****).

### Fluorescent tags can impact E2–E3 transthiolation

The interaction of either a UbcH7~Ub or UbcH5a~Ub conjugate with a HECT E3 ligase leads to Ub transfer from the E2~Ub conjugate to the E3 enzyme (Figure 1B). Since an E2 enzyme uses similar regions to interact with E1 and HECT E3 enzymes, we examined how fluorophore placement and size might impact Ub unloading from an E2~Ub conjugate. We formed E2~Ub conjugates *in situ* using Uba1, quenched these reactions using excess EDTA and measured single-turnover reactions for UbcH7~Ub and UbcH5a~Ub using the HECT domain of the E3 ligase HUWE1 (Figure 6). Both E2 enzymes were assessed using a large N-terminal YPet fluorophore or a small C-terminal AF488 fluorophore in conjunction with ^AF594^Ub. The make-up of these experiments precluded detailed kinetic measurements similar to those for E2~Ub conjugate formation. Both E2 enzymes show hyperbolic unloading profiles, monitored by the decrease of either YPet or AF594 fluorescence, upon addition of the HUWE1 HECT domain. Using UbcH7 as an example, the data also show minor amounts of hydrolysis (∼10%) over extended time periods and complete reduction of the conjugate with addition of excess DTT occur (Figure 6A). The two different UbcH7 enzymes E2 enzymes tested (^YPet^UbcH7, UbcH7^AF488^) show very similar unloading rates of ^AF594^Ub (Figure 6A), despite their distinctly different E2~Ub loading properties (Figures 3 and 4). In contrast, HUWE1-mediated unloading of ^AF488^UbcH5a∼^AF594^Ub is approximately 4-fold faster than observed for ^YPet^UbcH5a~^AF594^Ub indicating that for this E2 enzyme the larger GFP fluorophore impacts the Ub transfer to the HECT domain (Figure 6B,C).

**Figure 6:**
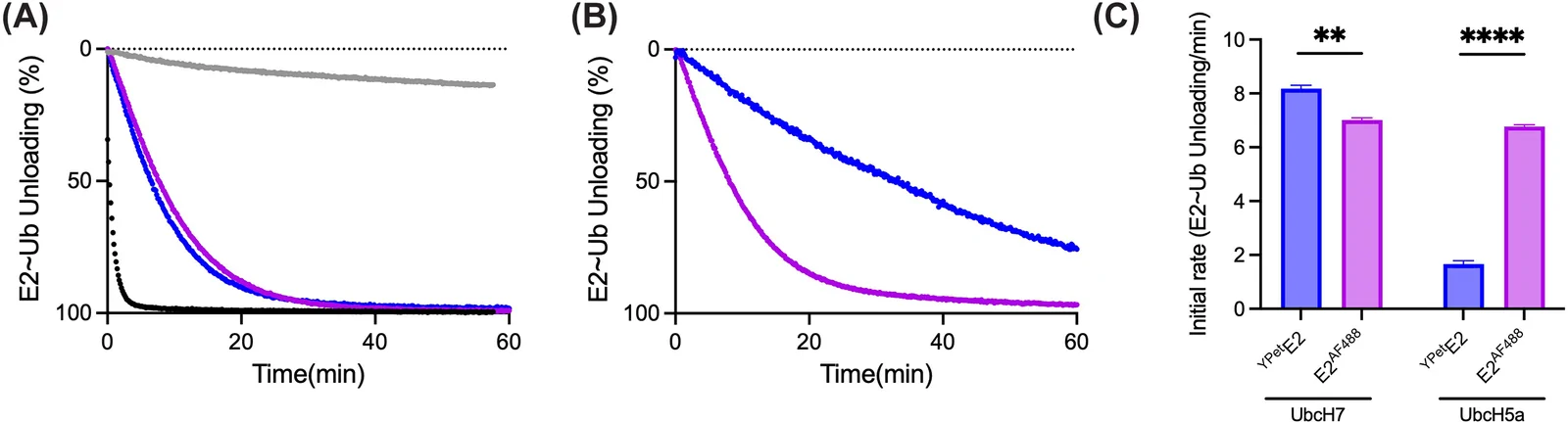
An N-terminal YPet tag impairs UbcH5a~Ub unloading to HUWE1 HECT domain E2~Ub unloading for FRET pairs using the HECT domain of HUWE1. (**A**) Normalized E2~Ub unloading curve for (A) ^YPet^UbcH7~^AF594^Ub (blue) or UbcH7^AF488^~^AF594^Ub (purple) and (**B**) ^YPet^UbcH5a~^AF594^Ub (blue) or UbcH5a^AF488^~^AF594^Ub (purple) with the HECT domain of HUWE1. In panel (A), a negative control (buffer only) shows minor hydrolysis (grey), whereas a positive control with 40 mM DTT shows complete hydrolysis of the E2~Ub conjugate (black). (**C**) Initial E2~^AF594^Ub unloading rates for UbcH7 and UbcH5a comparing ^YPet^E2 (blue) and E2^AF488^ (purple). Each bar is an average of three replicates with errors. A Student’s t-test was used to perform statistical analysis for *P-*values ≤ 0.01 (**) and 0.0001 (****).

## Discussion

Many *in vitro* and *in vivo* ubiquitination experiments utilize proteins that carry N-terminal or C-terminal appendages to optimize expression, solubility and detection. For example, GFP-based and antibody epitope tags have proven very useful to identify cellular localization or protein levels of the E1 enzyme Uba1 [59–62] and multiple E2 [63–67] and E3 enzymes [68–71], in addition to detection of ubiquitination efficiency and product formation. In this respect, the central E2 conjugating enzymes are unique because specific members of this protein family naturally carry extensions at the N- and/or C-termini that can have major roles in the functionality of the enzyme. The E2 enzyme UbcH6 (UBE2E1) has an N-terminal extension that inhibits its polyubiquitination activity [72], whereas CDC34 (UBE2R1) and E2–25K (UBE2K) have C-terminal extensions that control processivity such as chain elongation, linkage specificity and E3 interaction [73–75]. Additionally, structural studies show that the N-terminus of the core domain of an E2 enzyme engages with an E1 enzyme suggesting that appendages near this site could compromise enzymatic activity [21–24]. Despite these observations, there is limited information that shows how additional protein or small molecule tags on an E2 enzyme might compromise enzymatic activity.

In this work, we show that the size and location of a fluorescent reporter on an E2 enzyme can significantly influence the reaction rates for Ub transfer from an E1 to an E2 enzyme. We find that placement of a large GFP-type protein at the N-terminus of UbcH7, UbcH5a or Ubc13 perturbs Ub transfer to these E2 enzymes from Uba1. Our observations are supported by rate enhancements when placing a smaller Alexa-based dye at the N-terminus of UbcH7 or by moving the fluorophore (YPet or Alexa) to the C-terminus of the enzyme. These findings reinforce studies that show removal of a GST tag from the N-terminus of Ubc2b leads to a four-order increase in catalytic efficiency (*k*_cat_/*K*_m_) for transthiolation [25]. Alternatively, tagging E2–25K with an N-terminal GST tag results in *k*_cat_/*K*_m_ values around 80% that observed for the untagged protein, although this may be influenced by GST-driven dimerization that could alter ubiquitin chain formation [76]. Previously, experiments using ^YPet^UbcH5a have also shown that addition of the large YPet fluorophore can modify *k*_cat_ and *K*_m_ for Uba1-mediated Ub transfer [40] compared with the untagged protein [16,34]. The negative influence of modifications to the N-terminus of an E2 enzyme are also apparent through nanobody binding to the extreme N-terminus of CDC34 that delays Ub charging and reduces polyubiquitin chain formation [77]. These observations indicate that modifications made to the N-terminus of an E2 enzyme interfere with its interaction with the UFD, AAD and SCCH domains of Uba1 to compromise transthiolation. Nevertheless, all fluorescently labelled E2 and Ub proteins tested here were able to form E2~Ub conjugates over time. For many E2~Ub conjugation reactions, this measure may be sufficient. However, assays used to tease out minor differences in reactivity may benefit from shorter time-frame measurements and Alexa-fluor labelling of E2 and Ub proteins described here. One benefit of placing a small fluorophore such as AF488 at the C-terminus of an E2 enzyme is that it limits the impact on affinity for Uba1 compared to unmodified UbcH7. Reinforcing this, a C-terminal 6-His tag on the C-terminus of the E2 enzyme Ubc4 appears to have similar activity as the wild-type protein [24].

While not as dramatic, replacing the large CyPet fluorophore with a smaller AF594 dye at the N-terminus of Ub results in a 2.5–5-fold increase in the rates of UbcH7~Ub formation that is maximized with C-terminal labelling of UbcH7. In a similar manner, Wee *et al.* observed that labelling Ub at its N-terminus with the fluorophore Oregon green (similar size as AF488) shows a 10-fold increase in the Michaelis constant (*K*_m_) compared with unmodified Ub as a substrate for the human E1 enzyme Uba1 [39]. A further advantage of using the smaller Alexa-based dyes is their lack of dimerization that has been shown for both YPet and CyPet dyes (*K*_d_∼5 μM) in the concentration range for many *in vitro* ubiquitination reactions [78].

The transthiolation reaction from UbcH7~Ub to the HECT domain of the HECT E3 ligase HUWE1 did not show the same sensitivity for fluorophore placement and size as the E2 loading assays. However, an increased rate of unloading of UbcH5a~Ub conjugate was noted UbcH5a^AF488^ compared with the N-terminal YPet variant. This could be due to minor interaction differences with the HECT E3 ligase between the two E2 enzymes [48]. Alternatively the different reactivities for UbcH5a may reflect previous findings showing that reactivity of the isolated HECT domain of HUWE1 is diminished compared to the full length enzyme [49].

Our work provides a detailed analysis on the impact of modifying a subset of E2 enzymes with fluorescent tags and offers the ability to examine ubiquitination reactions in real time, with minimal steric or enzymatic interference. The use of smaller Alexa-based fluorophores has the potential for various applications, including measuring and comparing activity of different E2 variants, especially if small differences in catalytic efficiency are expected. This may be particularly applicable to high-throughput screening applications for small molecular inhibitors of E2 or E3 enzymes. It can be adapted to measure the unloading of E2~Ub conjugate in the presence of E2 substrates. Furthermore, there is scope to use these constructs to track the *in vivo* movement of E2 conjugates across various post-translational modification states.

## Methods

### Protein constructs and purification

Five different UbcH7 constructs were cloned from a base His_6_-TEV-UbcH7 (C17S, C137S) construct. UbcH7^YPet^ with a YPet tag in frame at the C-terminus, UbcH7 with an additional cysteine at the N-terminus (UbcH7 (C0)), and UbcH7 with an additional cysteine at the C-terminus (UbcH7 (C155)) were cloned into pET28 vectors using restriction-free cloning and site-directed mutagenesis, respectively. Two UbcH7 constructs were designed to contain only N- and C-terminal methionine residues. UbcH7 (M8L, M19L) with M1 at the N-terminus and UbcH7 (M1L, M8L, M19L) with M155 at the C-terminus were cloned into pET28 vectors using site-directed mutagenesis. pET28 plasmids containing a CyPet tag at the N-terminal of Ub (His_6_-^CyPet^Ub) or YPet tag at the N-terminus of UbcH5a (His_6_-^YPet^UbcH5a) were gifts from J. Liao (University of California, Riverside, CA). A YPet tag in frame with the N-terminus of UbcH7 (His_6_-^YPet^UbcH7) was cloned by replacing UbcH5a with UbcH7 in the His_6_-^YPet^UbcH5a plasmid using restriction-free cloning. A His_6_-TEV-^YPet^Ubc13 construct with YPet tag at its N-terminus and Ubc13 with an additional cysteine at the C-terminus [Ubc13 (C153)] were cloned from a base His_6_-TEV-Ubc13 construct using restriction-free cloning and site-directed mutagenesis, respectively. UbcH5a (C21A, C107A, C111S) with a C-terminal cysteine (C148) was cloned into a ProEx vector using site-directed mutagenesis. His_6_-TEV-Ub construct contains an additional cysteine at the N-terminus of Ub [His_6_-TEV-Ub (C0)]. The His_6_-TEV-HUWE1^4005-4374^ (HECT Domain) was acquired from Addgene (Plasmid #25211).

Uba1-His_6_, His_6_-Ub, His_6_-TEV-Ub (C0), His_6_-^CyPet^Ub, His_6_-TEV-UbcH7, His_6_-TEV-UbcH7 (C0), His_6_-UbcH7 (C155), His_6_-^YPet^UbcH7, His_6_-TEV-UbcH7^YPet^, His_6_-TEV-^YPet^Ubc13, Ubc13 (C153), His_6_-^YPet^UbcH5a, UbcH5a (C21A, C107A, C111S, C148) and His_6_-TEV-HUWE1^4005-4374^ constructs were expressed and purified using similar protocols. Constructs were transformed into BL21 (DE3) cells that were used to inoculate a 1L culture of Luria broth (LB) supplemented with appropriate antibiotic and grown at 37°C until the optical density at 600 nm (OD600) reached 0.6–0.8. Protein expression was induced by the addition of 0.5 mM (Uba1), 1 mM (Ub, Ub (C0), UbcH7, UbcH7 (C0), UbcH7 (C155), UbcH5a (C21A, C107A, C111S, C148), HUWE1^4005-4374^), 0.2 mM (^CyPet^Ub, ^YPet^UbcH7, ^YPet^UbcH5a, UbcH7^YPet^), 0.4 mM (^YPet^Ubc13, Ubc13 (C153)) isopropyl β-d-1-thiogalactopyranoside (IPTG) and the temperature of the culture was lowered to 30°C for UbcH7 constructs, 25°C for CyPet and YPet constructs or 16°C for other constructs for overnight growth. Ubc13 (C153) was grown for 5 h at 37°C after induction. The cells were harvested, resuspended in lysis buffer [UbcH7, UbcH5a, Ubc13–25 mM Tris (pH 7.5), 500 mM NaCl, 5% glycerol; Ub, Uba1 and HUWE1 constructs – 25 mM Tris (pH 8), 300 mM NaCl] and lysed using an EmulsiFlex-C5 homogenizer (Avestin). The proteins were purified using Ni^2+^-NTA affinity chromatography. The His_6_ tag was removed using Tobacco Etch Virus (TEV) protease for all constructs except Uba1-His_6_, His_6_-^CyPet^Ub, His_6_-^YPet^UbcH5a, His_6_-TEV- ^YPet^Ubc13 and His_6_-^YPet^UbcH7 and cleaved proteins were isolated using reverse Ni^2+^-NTA chromatography. Fractions containing protein were analyzed by SDS–PAGE and concentrated. His_6_-^CyPet^Ub, His_6_-^YPet^UbcH7, and UbcH7^YPet^ were further purified using a gel filtration column (HiLoad 16/60 Superdex75pg, Cytiva) and an anion-exchange column (HiTrap Q XL (strong anion), Cytiva). HUWE1^4005-4374^ was further purified using an anion exchange. All proteins were confirmed by mass spectrometry (SPARC Biocentre at the Hospital for Sick Children, Toronto, Canada).

The His_6_-TEV-UbcH7 (M8, 19L) and His_6_-TEV-UbcH7 (M1L, M8L, M19L, 155M) were transformed into B834 (DE3) methionine auxotrophic cells. A single colony was used to inoculate an overnight culture at 37°C in selective Luria broth (LB) (30 μg/ml kanamycin). This culture was used to inoculate a larger 1 l batch of selective minimal medium containing 0.04 mM methionine to achieve a starting OD600 of 0.1 [79]. The cells were grown at 37°C until they reached an OD600 of 0.9–1.0, followed by incubation on ice for 30 min. 0.5 mM AHA was added, and the cells were recovered for 30 min at 37°C followed by induction with 1 mM IPTG and grown overnight at 25°C. The protein was purified similarly to the His_6_-TEV-UbcH7 construct. All purified proteins were dialyzed into 50 mM HEPES (pH 7.5), 250 mM NaCl, and 1 mM DTT, and flash frozen for storage at −80°C.

### Chemical labelling of proteins

#### Preparation of ^AF594^Ub

Ub (C0) was dialyzed into 50 mM sodium phosphate (pH 7.2), 100 mM NaCl, 1 mM EDTA and 1 mM TCEP. Alexa Fluor^™^ 594 C_5_ maleimide (Invitrogen) was added at a 1:1 ratio to Ub (C0) and incubated at room temperature for 30 minutes, followed by quenching with excess 5 mM DTT. The reaction mixture was purified on a gel filtration column (Superdex75 10/300 GL column, Cytiva) to separate unreacted dye from the labelled protein. The ^AF594^Ub protein was stored in 50 mM HEPES (pH 7.5), 250 mM NaCl, and 1 mM DTT.

#### Preparation of ^AHA*AF488^UbcH7 and UbcH7^AHA*AF488^

100 μM UbcH7 (M8,19L), and UbcH7 (M1L, M8L, M19L, 155M) were incubated with 250 μM 5,5′-dithiobis-(2-nitrobenzoic acid) (DTNB) for 10 min to block the catalytic cysteine from reacting with alkyne group of AF488 Dibenzocyclooctyne (DBCO) dye (Lumiprobe). 500 μM AF^488^ DBCO dye was added and incubated for 1 h at room temperature with gentle rocking. The reaction was centrifuged to remove any precipitate and passed through a gel filtration column (Superdex75 10/300 GL column, Cytiva) using 50 mM HEPES (pH 7.5) and 250 mM NaCl buffer to remove excess dye and DTNB. 5 mM DTT was added to reduce the catalytic cysteine, and the protein was dialyzed into 50 mM HEPES (pH 7.5), 250 mM NaCl, and 1 mM DTT for storage.

#### Preparation of ^AF488^UbcH7 and UbcH7^AF488^

200 μM UbcH7 (C0) and UbcH7 (C155), respectively, were incubated with 25 μM Uba1 and 1.5 mM His-Ub at 37°C for 30 min in 50 mM HEPES (pH 7.5) and 2 mM TCEP. After equilibration, E2~Ub conjugate formation was initiated with the addition of 5 mM MgATP and incubated for 30 min. 1.25 mM and 2.5 mM AF488-maleimide dye (Lumiprobe) was added to UbcH7 (C0) and UbcH7 (C155), respectively, and the reactions were incubated for 30 min at room temperature with gentle rocking. Centrifugation was used to remove any precipitate, and samples were passed through a gel filtration column (HiLoad 16/60 Superdex75pg column, Cytiva) using 50 mM Tris (pH 6.0) and 250 mM NaCl buffer to separate E2~Ub conjugate from other proteins (Uba1, unreacted UbcH7 and His-Ub) and excess dye. 4 mM TCEP was added to reduce the E2~Ub conjugate, and the protein was dialyzed into 50 mM Tris (pH 7.5), 250 mM NaCl, 1 mM DTT and 250 μM TCEP overnight. The AF488−labelled UbcH7 proteins were separated from the His_6_-Ub using Ni^2+^-NTA affinity chromatography and stored in 50 mM Tris (pH 7.5), 250 mM NaCl, 1 mM DTT and 250 μM TCEP. UbcH5a^AF488^ and Ubc13^AF488^ were prepared using similar methodology.

### Gel based-E2~Ub loading assays

0.5 μM Uba1, 10 μM UbcH7 constructs and 20 μM Ub constructs were incubated at 27°C for 15 min in 50 mM HEPES (pH 7.5) and 500 μM TCEP. The conjugation reaction was initiated with the addition of 5 mM MgATP and quenched at desired time points using acidified 3× SDS sample buffer (0.001% TFA). Samples were analyzed by SDS–PAGE using a gradient gel (4–12% Bis–Tris Plus, Thermo Fisher Scientific) and MES running buffer (50 mM MES, 50 mM Tris, 0.1% SDS, 1 mM EDTA, pH 7.3) and stained using Coomassie blue for visualization.

### FRET E2~Ub loading assays

E2 loading assays were conducted in triplicate using equimolar amounts of E2 and Ub proteins (0.1–4 μM range), 5 nM Uba1, 50 mM HEPES (pH 7.5), 500 μM TCEP and 0.1% BSA. Samples were measured using black, non-binding surface (NBS), half-area 96-well plates with clear flat bottoms (Corning) using a Synergy H1 microplate reader controlled by Gen5 software (Biotek, Winooski, VT). The system was temperature set to 27°C, and the instrument was operated in fluorescence end-point mode using a xenon flash lamp. A system gain of 100 in extended gain mode with a bottom read setting was used for all measurements. Two kinetic loops were used for data collection with 10 s time points: (i) 15 min equilibration, and (ii) 10 min to 1 h collection. 5 mM MgATP was added for reaction initiation before the start of the second kinetic loop. The donor fluorophore was excited, and the acceptor emission was measured at the appropriate wavelength for each FRET pair (Supplementary Table S1). Data collected from the second kinetic loop were normalized for background fluorescence using the equilibration fluorescence from the first kinetic loop. The initial rate of E2~Ub conjugate formation was calculated from the first 90 s of data points after MgATP addition. Conversion factors were calculated to convert FRET signal (RFU) to the amount of product formed (moles) from the slope of the maximum FRET signal plotted against the known concentration of conjugate formed for each FRET pair. Initial rates were fit to the Michaelis–Menten equation to derive the kinetic parameters (*K*_m_ and *k*_cat_). GraphPad Prism was used to conduct all statistical analyses and data fitting.

### Competitive inhibition assays

The assays were conducted in triplicate using 1 μM E2 and 2 μM Ub FRET constructs with a concentration range of 0–50 μM unlabeled UbcH7, 5 nM Uba1, 50 mM HEPES (pH 7.5), 500 μM TCEP and 0.1% BSA. The data were collected using Synergy H1 microplate reader as described above. The initial rates of E2~Ub formation were normalized to the rate at 0 μM unlabeled UbcH7. The data were fit for competitive inhibition using the Cheng–Prusoff equation to derive the kinetic inhibition parameter (*K*_i_) [80]. In the equation, *ν*_0_ and ν*ν*_i_ represent the initial rate of E2~Ub conjugate formation in the absence and presence of unmodified UbcH7, respectively, *I* is the concentration range of unmodified UbcH7 (0–50 μM), S is the concentration of fluorescently-labelled UbcH7 constructs (1 μM), and *K*_m_ is the Michaelis constant for FRET UbcH7 constructs and Uba1 affinity.

$$\frac{v_{0}}{v_{i}}=\frac{1}{1+\left([I]/K_{i}\left(1+(S/K_{m})\right)\right)}$$

### E2~Ub unloading assays

E2~Ub conjugates were formed *in situ* using 50 nM Uba1, 1 μM E2 and 2 μM Ub in 50 mM HEPES (pH 7.5), 500 μM TCEP and 0.1% BSA. Four kinetic loops were used for data collection collecting data points every 10 s: (i) 5 min for equilibration, (ii) 30 min after addition of 5 mM MgATP for E2~Ub conjugate formation, (iii) 5 min after addition of 20 mM EDTA to quench Uba1 activity, and (iv) 1–2 h after addition of 10:1 ratio of HUWE1:E2 to measure unloading of the E2~Ub conjugate. The data were collected using Synergy H1 microplate reader as described above.

## Supplementary Material

Supplementary Figures S1-S3 and Table S1

## Data Availability

All data are shown in the manuscript. All reagents and protein constructs are available from the authors upon request.

## Open Access

Open access for this article was enabled by the participation of the University of Western Ontario in an all-inclusive Read & Publish agreement with Portland Press and the Biochemical Society under an agreement with CAUL.

## Abbreviations

AHA: azidohomoalanine
PTM: post-translational modification
UFD: ubiquitin-fold domain
Ub: ubiquitin
