# Supplementary material for: Fluorophore placement and size impact E2 ubiquitination efficiency
Source: Biochem J. 2026 Oct 8;483(11):2091–106. doi: 10.1042/BCJ20260496 (PMC13660846; doi:10.1042/BCJ20260496)
Supplement: Supplementary file 1 [file BCJ-2026-0496_supp.pdf]

SUPPLEMENTAL FIGURES

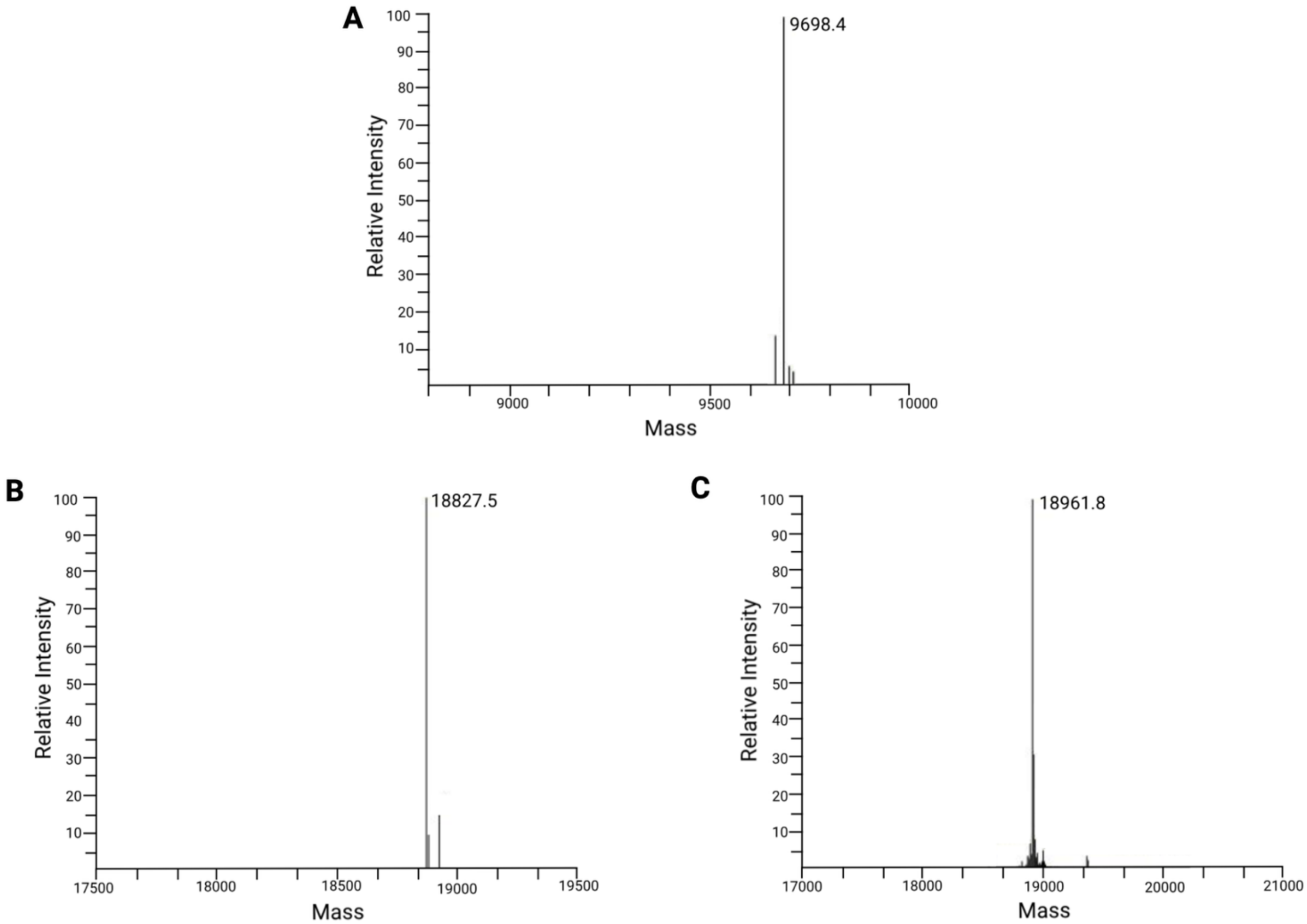

Figure S1: ES1 mass spectra of (A) ^{AF594}Ub with an expected mass of 9700 Da, (B) UbcH7^{AF488} with an expected mass of 18827 Da and (C) ^{AF488}UbcH7 with an expected mass of 18827 Da.

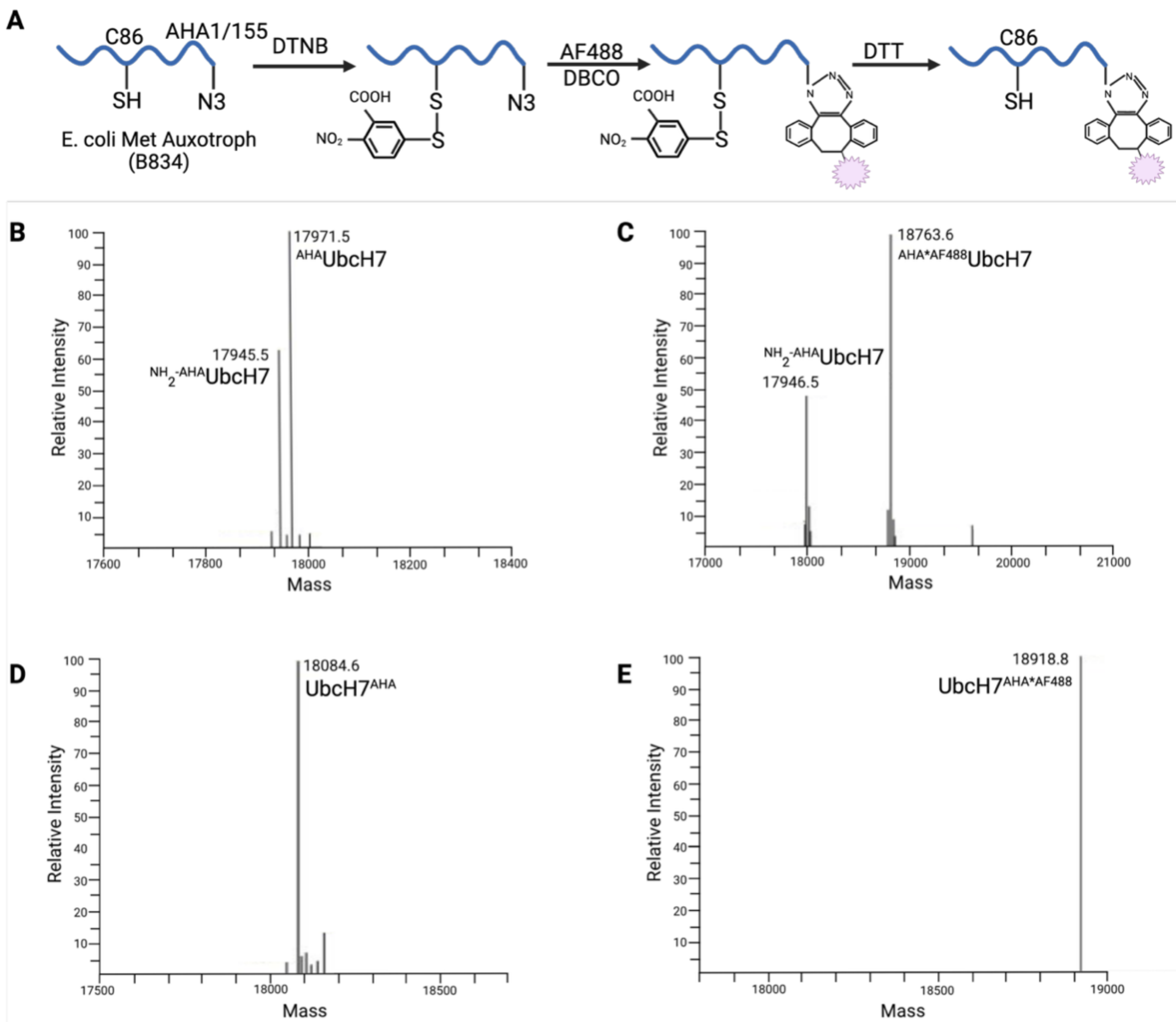

Figure S2: (A) UbcH7 labelling strategy using cycloaddition-based copper-free click chemistry. UbcH7 constructs with M1 at the N-terminus (M8L, M19L) and M155 at the C-terminus (M1L, M8L, M19L) were used. Constructs were transformed into a Met-auxotrophic strain of *E. coli* (B834) and supplemented with excess Azidohomoalanine (AHA). Purified proteins were treated with 5,5'-dithiobis-(2-nitrobenzoic acid) (DTNB) to block the catalytic cysteine (C86) followed by AF488 Dibenzocyclooctyne (DBCO) dye. The labelled proteins were reduced with DTT to obtain AF488 labelled UbcH7 proteins. ESI mass spectra of (B) UbcH7^{AHA} (expected mass 18085 Da), (C) UbcH7^{AHA*AF488} (expected mass 18876 Da), (D) ^{AHA}UbcH7 (expected mass 17972 Da), and (E) ^{AHA*AF488}UbcH7 (expected mass 18763 Da).

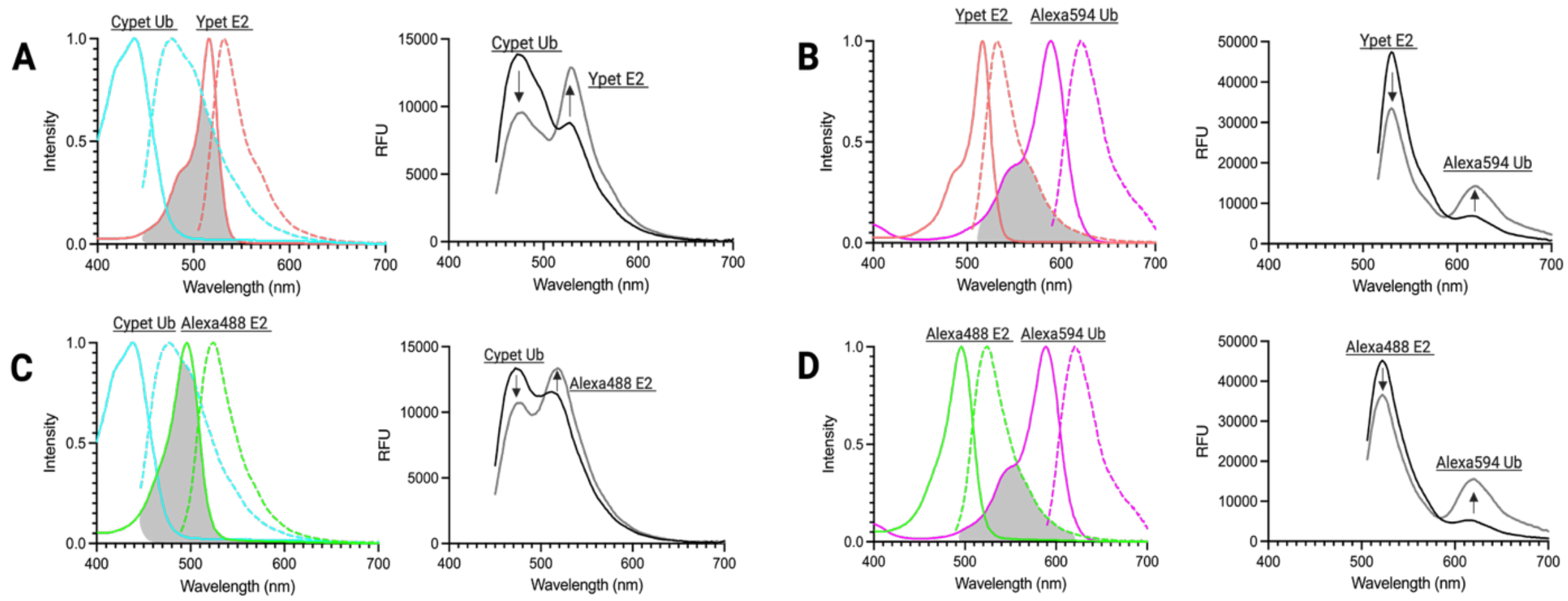

Figure S3: Measured spectral profiles (left panel) and emission spectra (right panel) for E2 and Ub FRET pairs (donor and acceptor). Measured spectra are shown for (A) ^{CyPet}Ub (cyan) and ^{YPet}UbcH7 (orange), (B) ^{YPet}UbcH7 (orange) and ^{AF594}Ub (magenta), (C) ^{CyPet}Ub (cyan) and UbcH7^{AF488} (green), and (D) UbcH7^{AF488} (green) and ^{AF594}Ub (magenta). Left Panel: All spectral scans were done for the real-time E2 loading assay with an equimolar ratio of Ub to E2 and were normalized to 1 internally. The shadow area represents FRET overlap between the donor emission spectra and acceptor excitation spectra. Right panel: Emission spectra were measured at time 0 (black line) and 10 minutes (grey line) following initiation of E2~Ub conjugate formation upon addition of ATP.

Table 1: Summary of E2 and Ub FRET pairs with their respective donor and acceptor excitation and emission wavelengths.

**FRET Pair**	**Donor**	**Acceptor**	**Excitation (nm)**	**Emission (nm)**
^{YPet}UbcH7 - ^{CyPet}Ub	CyPet	YPet	420	528
UbcH7^{YPet} - ^{CyPet}Ub	CyPet	YPet	420	528
^{YPet}UbcH7/ ^{YPet}UbcH5a/^{YPet}Ubc13 – ^{AF594}Ub	YPet	AF594	490	620
UbcH7^{YPet} – ^{AF594}Ub	YPet	AF594	490	620
^{AF488}UbcH7 - ^{CyPet}Ub	CyPet	AF488	420	520
^{AF488}UbcH7- ^{AF594}Ub	AF488	AF594	480	620
UbcH7^{AF488} - ^{CyPet}Ub	CyPet	AF488	420	520
UbcH7^{AF488}/UbcH5a^{AF488}/Ubc13^{AF488}-^{AF594}Ub	AF488	AF594	480	620
